# Broadband diffuse optical spectroscopy of absolute methemoglobin concentration can distinguish benign and malignant breast lesions

**DOI:** 10.1117/1.JBO.26.6.065004

**Published:** 2021-06-29

**Authors:** Sandhya Vasudevan, Chris Campbell, Fang Liu, Thomas D. O’Sullivan

**Affiliations:** a University of Notre Dame, Department of Electrical Engineering, Notre Dame, Indiana, United States; b University of Notre Dame, Department of Applied and Computational Mathematics and Statistics, Notre Dame, Indiana, United States

**Keywords:** diffuse optical imaging, near-infrared spectroscopy, breast cancer, tissue optics, methemoglobin, spectroscopy

## Abstract

**Significance:** Noninvasive diffuse optical spectroscopy (DOS) is a promising adjunct diagnostic imaging technique for distinguishing benign and malignant breast lesions. Most DOS approaches require normalizing lesion biomarkers to healthy tissue since major tissue constituents exhibit large interpatient variations. However, absolute optical biomarkers are desirable as it avoids reference measurements which may be difficult or impractical to acquire.

**Aim:** Our goal is to determine whether absolute measurements of minor absorbers such as collagen and methemoglobin (metHb) can successfully distinguish lesions. We hypothesize that metHb would exhibit less interpatient variability and be more suitable as an absolute metric for malignancy. However, we would expect collagen to exhibit more variability, because unlike metHb, collagen is also present in the healthy tissue.

**Approach:** In this retrospective clinical study, 30 lesions with breast imaging reporting and database system score (BIRADS)>=3 (12 benign and 18 malignant) measured with broadband quantitative DOS were analyzed for their oxyhemoglobin (HbO), deoxyhemoglobin (HHb), water, lipids, collagen, metHb concentrations, and optical scattering characteristics. Wilcoxon rank sum test was used to compare benign and malignant lesions for all variables in both normalized and absolute forms.

**Results:** Among all absolute DOS parameters considered, only absolute metHb was observed to be significant for lesion discrimination (0.43±0.18  μM for benign versus 0.87±0.32  μM for malignant, p=0.0002). Absolute metHb concentration was also determined to be the best predictor of malignancy with an area under the curve of 0.89.

**Conclusions:** Our findings demonstrate that lesion metHb concentration measured by DOS can improve noninvasive optical diagnosis of breast malignancies. Since metHb concentration found in normal breast tissue is extremely low, metHb may be a more direct indicator of malignancy that does not depend on other biomarkers found in healthy tissue with significant variability. Furthermore, absolute parameters require reduced measurement time and can be utilized in cases where healthy reference tissue is not available.

## 1 Introduction

Breast cancer is one of the most common cancers worldwide and is the second leading cause of cancer death among women.^1^ Breast cancer screening via mammography is recommended to detect and treat breast cancers at an earlier stage, which dramatically improves survival.^2^ However, mammography is less accurate in younger women and individuals with radiographically dense breasts.^3^^,^^4^ In addition, mammography results in a significant number of false positive callbacks and biopsies. For every breast cancer detected by screening mammography, approximately three patients undergo biopsy, among which two biopsies will be benign.^5^ In women with dense breast tissue, supplemental ultrasound increases the number of cancers detected but doubles the number of biopsies that are ultimately negative.^6^^,^^7^ These additional biopsies are often associated with increased anxiety, pain, bruising, scarring, and cost. Several studies have also noted increased distress and other psychological effects as a result of false positives in women.^8^^,^^9^

Noninvasive optical-based tissue sensing and imaging using diffuse optical spectroscopy (DOS), a form of near-infrared spectroscopy, measures functional and metabolic information of deep tissue (up to 2 to 3 cm) using red and near-infrared light. DOS provides a molecular analysis of the tissue through its measurement of optical absorbers and has been used extensively to evaluate breast lesion composition in clinical research settings.^10^ An elevated relative concentration of hemoglobin, compared with surrounding (or contralateral) normal tissue, has been shown to be a good discriminator for cancer. This reflects the higher and abnormal vascularity typically found in solid malignancies.^11^^–^^19^ Several studies have also shown a reduction of lipid content and an increase of water and blood in breast tumors compared with normal breast tissue,^10^^,^^20^^–^^22^ while the structural protein collagen can be elevated in malignant breast lesions due to the correlation of collagen deposition and cross linking to tumor development and progression.^23^^–^^27^ Scattering parameters, which characterize overall cellular density and size, have also been shown to be significantly higher in malignant compared with benign lesions.^21^^,^^28^ The presence of spectral contributions to overall tumor absorption that are not accounted for by the major tissue absorbers such as oxyhemoglobin (HbO), deoxyhemoglobin (HHb), water, and bulk lipids have been reported by DOS studies.^29^ These “specific tumor components (STC)” have also been shown to be clinically significant in differentiating between benign and malignant lesions.^20^^,^^21^^,^^29^^,^^30^

Most DOS approaches require normalization of the lesion values to healthy breast tissue since major tissue absorbers such as blood, water, and fat exhibit large normal interpatient variations^31^^,^^32^ due to diverseness in age, menstrual cycle, and hormonal status of the patients. In this study, we investigated the role of the absolute tissue concentrations of minor optical absorbers such as collagen and metHb in breast cancer diagnosis.^33^ We hypothesize that metHb would endure less interpatient variability and be more suitable as an absolute metric for malignancy. However, we would expect collagen to exhibit more interpatient variability, because unlike metHb, collagen is also present in the normal healthy breast tissue. In this study, the *in-vivo* characterization of benign and malignant breast lesions in 28 patients (30 lesions) is reported in terms of HbO, HHb, water, lipids, collagen, metHb, and scattering parameters. To the best of our knowledge, this is the first work that reports metHb as a potential biomarker for breast lesion characterization. The long-term goal of this work is to improve differential diagnosis of breast lesions in dense breasts and to reduce the overall false-positive rate in the detection of breast cancer.

## 2 Materials and Methods

### 2.1 Patients and Study Design

We performed a retrospective analysis of broadband quantitative DOS data collected from a mixed cohort of pre- and postmenopausal subjects (28 patients) with 30 suspicious breast lesions before biopsy (12 benign and 18 malignant), among which most lesions (21 out of 30) were categorized BIRADS 3 or 4.

Table 1 presents the characteristics of the subjects. The average age of subjects was 47 (age range, 20 to 73 years). Within each group, mean age and range were as follows: malignant group (mean age, 53 years; range, 37 to 73 years) and benign group (mean age, 37 years; range, 20 to 50 years).

**Table 1:** Subject characteristics.

Variable		All cases (n=28)	Malignant cases (n=18)	Benign cases (n=10)
Mean age (min–max)	(years)	47 (20–73)	53 (37–73)	37 (20–50)
Menopausal status (frequency)	Premenopausal	19	10	9
	Postmenopausal	8	7	1
	Perimenopausal	1	1	0

Table 2 reports the lesion characteristics at the subject level. The average (SD, range) lesion size measured by ultrasound (US) imaging (maximum dimension) was 1.99 cm (1.09 cm, 0.70 to 5.60 cm). The average (SD, range) lesion depth measured by US imaging was 0.82 cm (0.38 cm, 0.10 to 1.60 cm).

**Table 2:** Lesion characteristics.

(No. of lesions)	All cases (30)	Malignant cases (18)	Benign cases (12)
Maximum US size	Mean±SD; range (cm)		
	1.99±1.09	2.23±1.23	1.63±0.76
	0.70−5.60	1.00−5.60	0.70−2.80
US depth	Mean±SD (range) (cm)		
	0.82±0.38	0.76±0.36	0.90±0.41
	0.10−1.60	0.10−1.30	0.30−1.60
BIRADS classification	Frequency		
	7	0	7
4	14	9	5
5	4	4	0
6	5	5	0
Lesion type	Frequency		
IDC	—	13	—
DCIS	—	1	—
IDC + DCIS	—	4	—
Fibroadenoma	—	—	9
Fibrocystic change	—	—	1
Adenomyoepithelioma	—	—	1
Oil cyst	—	—	1

### 2.2 DOS Data Acquisition

These data were originally collected for a prospective study of hybrid frequency-domain and continuous-wave broadband DOS for breast cancer diagnosis.^21^ Though the prior analysis included broadband data from 650 to 1000 nm, there was additional useful data down to 640 nm. Therefore, we analyzed DOS absorption from 640 to 1000 nm to attain a more accurate quantification of methemoglobin. The technical details of the DOS system are described elsewhere.^34^^,^^35^ Briefly, four laser diodes (660, 690, 780, and 830 nm) were sequentially swept from 50 to 500 MHz to acquire multifrequency amplitude and phase measurements using an avalanche photodiode in contact with the tissue surface. The CW component consists of a tungsten–halogen white light source and a fiber coupled to the spectrometer to measure the broadband reflectance spectra. FD and CW data were collected from an overlapping volume of tissue using a handheld probe with a source–detector separation of 28 mm.

### 2.3 Data Preprocessing

Broadband absorption ($\mu_{a}$) and reduced scattering ($\mu_{s}^{\prime }$) spectra in the wavelength range of 640 to 1000 nm were acquired by reprocessing FD and CW raw data acquired in the previous study.^21^ FD data at a continuous range of modulation frequencies, calibrated using a tissue-simulating phantom, was fit to a p1 diffusive model of light transport with semi-infinite boundary conditions. The end frequency was selected on a subject-by-subject basis based on signal to noise-ratio and varied from 350 to 500 MHz. The initial frequency was set to 50 MHZ in all cases. Details of the broadband DOS technique that combines FD and CW data to produce broadband $\mu_{a}$, $\mu_{s}^{\prime }$ spectral results are provided in Ref. 35. Briefly, the reduced scattering coefficients $\mu_{s}^{\prime }$ at the FD wavelengths were used to estimate tissue optical scattering amplitude (a) and power (b) by fitting the data to the empirical Mie scattering relationship $\mu_{s}^{\prime }(\lambda )=a{\left(\lambda /\lambda_{0}\right)}^{-b}$, where $\lambda_{0}=500\;\mathrm{nm}$.^36^^,^^37^ $\mu_{s}^{\prime }$ was estimated for each CW wavelength from the recovered a and b values for wavelengths in the spectral range 640 to 1000 nm. Finally, the broadband $\mu_{s}^{\prime }(\lambda )$ were used to provide a scatter correction for the CW reflectance measurements to extract the absolute broadband absorption spectrum $\mu_{a}(\lambda )$.

**Fig. 1:**
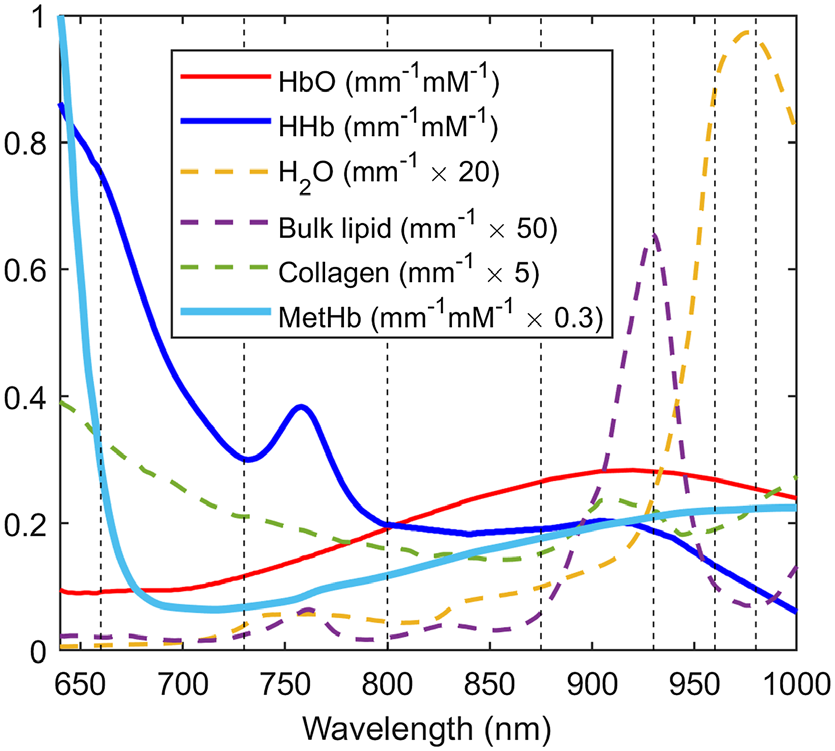
Extinction spectra of tissue absorbers.

Concentrations of HbO, HHb, water, lipids, collagen, and methemoglobin were calculated by fitting a linear combination of their known molar extinction coefficient spectra^38^^–^^40^ (Fig. 1) to the tissue absorption spectra (640 to 1000 nm) via an ordinary unconstrained least squares curve fit.^41^ The quality of the fit of the individual absorbers to the tissue absorption spectra was evaluated by the adjusted R squared ($R_{a}^{2}$). $R_{a}^{2}$ determines the fit quality while considering for the number of variables (absorbers) included in the model.^42^ A potential downside of adding more chromophores is coincidental improvement of the spectral fit which can be misleading. Therefore, we have calculated $R_{a}^{2}$ that penalizes model complexity to evaluate whether the minor absorbers (collagen and metHb) are useful variables for the model.

**Fig. 2:**
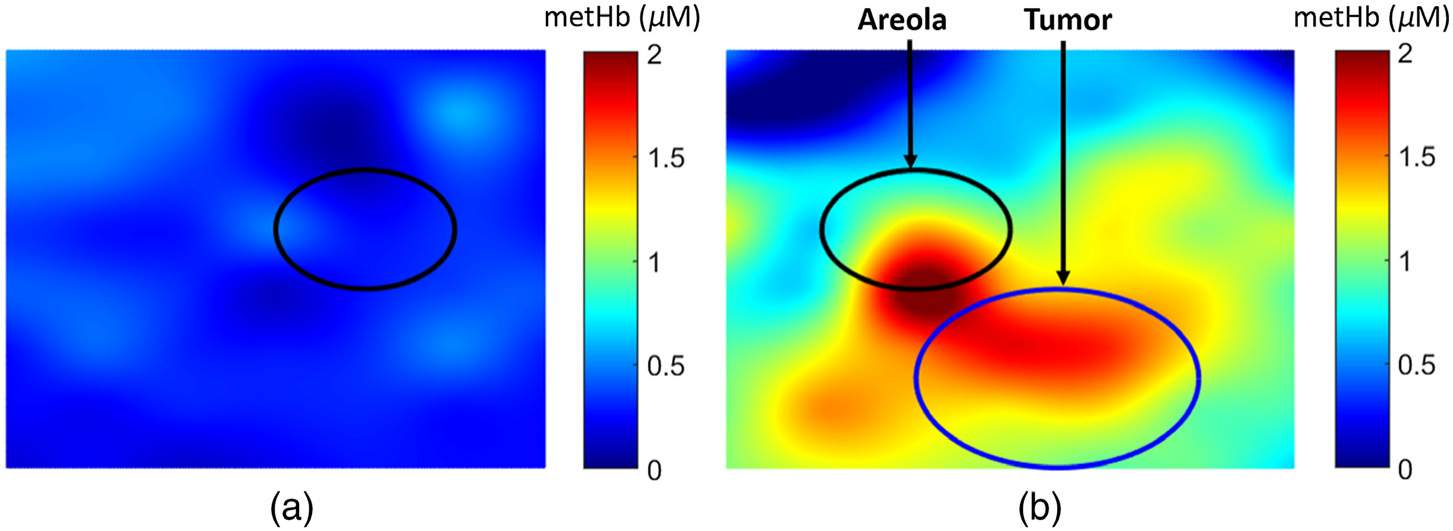
DOS image of metHb concentration on (a) normal left breast and (b) right breast with a 15-mm radius malignant lesion.

For each patient, chromophore concentrations and scattering parameters were measured from locations on both lesion-containing breast (lesion side) and contralateral breast (normal side). From the DOS images, a region of interest (ROI) representing the lesion was defined on each patient using the US lesion size and was centered on the tissue optical index ($\mathrm{TOI}=\frac{\mathrm{HHb}\times \text{water}}{\text{lipids}}$) lesion enhancement. The lesion ROI was mirrored on the contralateral breast to define the normal tissue ROI. Lesion and normal DOS variables (chromophore concentrations and scattering parameters) were determined by calculating the mean and standard deviation of the measurement locations within the ROIs. In this work, we investigated both the absolute values as well as the lesion-to-normal ratio (L/N) of the DOS variables in differentiating benign and malignant lesions. Quantitative images of metHb concentration have been provided in Fig. 2 for visualization.

### 2.4 Statistical Analysis

The Wilcoxon rank sum test was used to compare benign and malignant lesions for each of the eight biomarkers. Statistical significance was claimed for p-value <0.00625, where the significance threshold has been corrected for multiplicity using the Bonferroni method at the overall significance level of 0.05 for testing eight hypothesis simultaneously (i.e., 0.05/8=0.00625). The area under the curve (AUC) along with 95% confidence interval was also calculated for each of the eight DOS variables considered. We report accuracy, sensitivity, specificity, negative predictive values (NPVs), and positive predictive values (PPVs) for all DOS variables at a threshold that demonstrates the minimum difference between sensitivity and specificity.

Because most of the postmenopausal subjects (seven out of eight) in the dataset exhibited malignant lesions, multiple linear regression was utilized to analyze benign versus malignant lesion differentiation power of each absolute parameter, correcting for the baseline characteristics including age and menopausal status. For this analysis, each of the eight absolute variables was the response variable and was log-transformed to better satisfy the normality assumption of the regression: lesion category (benign and malignant), age, and menopausal status (pre- and postmenopausal) were considered as predictor variables. Given the data sparsity in the perimenopausal subjects (n=1), peri- and postmenopausal cases were aggregated into one group. For the regression analysis, we report the coefficient estimates, the associated standard errors, p-values for the predictors in the model. For the regression analysis, statistical significance was assumed for p-value <0.05. All statistics were performed via Statistics and Machine Learning toolbox, MATLAB 2019b.

## 3 Results

### 3.1 Extracting Minor Absorbers

**Fig. 3:**
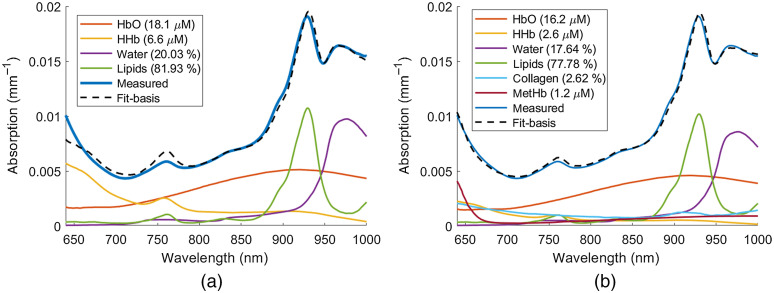
Absorption spectrum of a breast lesion, expressed as the sum of absorption spectra of the (a) basis absorbers and (b) basis and minor absorbers. The measured values of the absorption coefficient spectra are also reported. The estimated concentrations of all absorbers have been included.

An example of a lesion absorption spectrum, expressed as the sum of absorption spectra of the basis absorbers (HbO, HHb, water, and lipids) as well as minor absorbers collagen and methemoglobin is shown in Fig. 3(b). For comparison, the absorption spectrum and constituent components without the minor absorbers are shown in Fig. 3(a). Figure 3(a) shows the conventional technique adopted in the previous study^21^ where only contribution from the major absorbers (HbO, HHb, water, and lipids) were used to characterize the lesion absorption. Root mean squared error (RMSE) and adjusted R squared ($R_{a}^{2}$) values of the spectral fits illustrated in Figs. 3(a) and 3(b) are summarized in Table 3.

**Table 3:** Spectral fit parameters.

	Basis absorbers	Basis and minor absorbers
RMSE	$4.87\times 10^{-4}$	$2.4\times 10^{-4}$
$R_{a}^{2}$	0.9885	0.9972

Figure 3(a) and 3(b) show that the addition of collagen and methemoglobin improves the quality of the spectral fit of the absorbers to the measured lesion absorption spectrum. The wavelength regions that exhibit improvement of the absorption fit with inclusion of minor absorbers are 640 to 780 nm and 880 to 1000 nm. The corresponding decrease in RMSE and increase in $R_{a}^{2}$ values (Table 3) validates the improvement of the linear model with the inclusion of the additional chromophores.

### 3.2 Optical Differences between Benign and Malignant Lesions

#### 3.2.1 Normalized lesion to normal (L/N) parameters

**Fig. 4:**
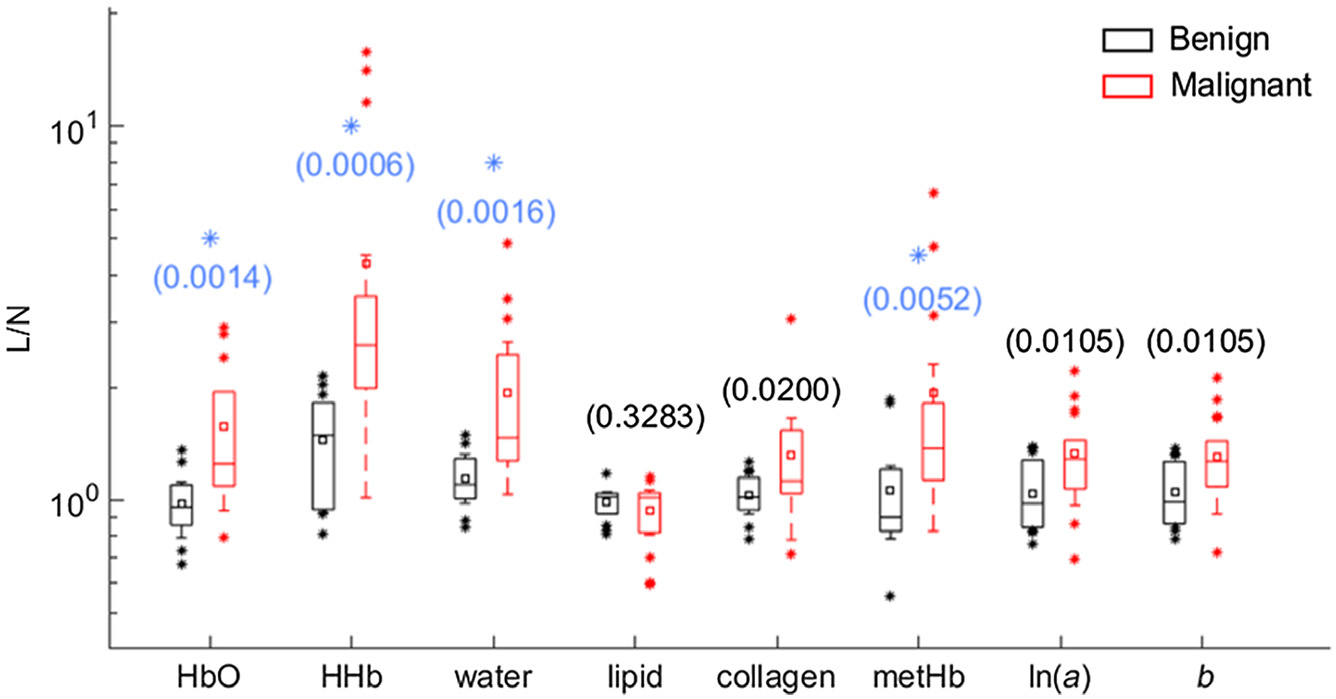
Box plots of L/N in HbO, HHb, water, lipid, collagen, metHb, ln(a), and b in the groups of benign and malignant lesions. Mean value in each group is indicated with a square. Boxes show the median and the 25th and 75th percentiles. Whiskers show the SD, and * represents the outliers. The blue * indicates a statistically significant difference between the two groups from the Wilcoxon rank sums test with the Bonferroni correction at α=0.05.

The distribution of L/N values in DOS parameters for the benign and malignant cases are summarized in Fig. 4. Malignant lesions exhibited larger mean L/N ratio than the benign group for all DOS variables except for lipid. These differences were found to be statistically significant (p<0.00625) for HbO, HHb, water, and metHb L/N values per the Wilcoxon rank sum test with correction for multiple comparisons.

**Fig. 5:**
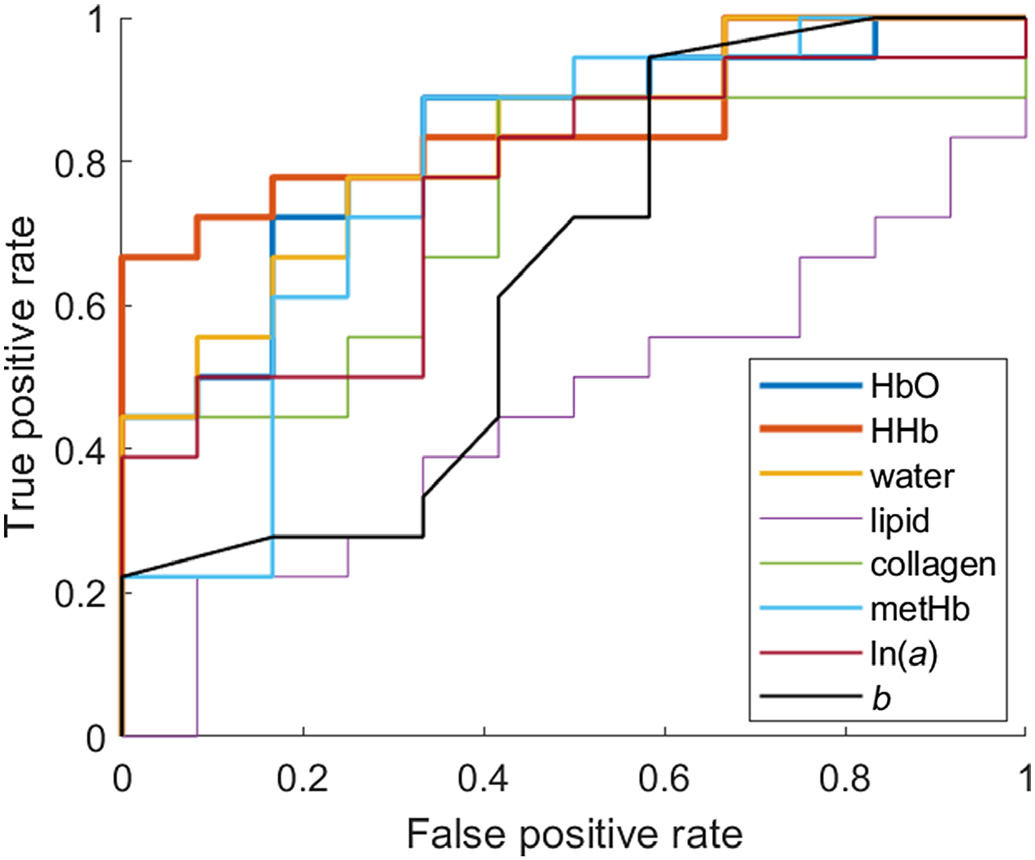
ROC curve of L/N values of DOS parameters.

The area under the ROC curve (AUC) of all L/N parameters are shown in Fig. 5 and Table 4. Accuracy, sensitivity, specificity, NPVs, and PPVs of the L/N parameters at an optimum threshold have also been summarized in Table 4.

**Table 4:** AUC, 95% confidence interval and accuracy, sensitivity, specificity, PPV, and NPV at the specified threshold for L/N parameters.

L/N parameter	AUC (95% CI)	Threshold	Accuracy	Sensitivity	Specificity	PPV	NPV
HbO	0.83 (0.63 to 0.94)	1.09	76.7	77.8	75.0	82.4	69.2
HHb	0.86 (0.68 to 0.96)	1.91	76.7	77.8	75.0	82.4	69.2
Water	0.82 (0.62 to 0.95)	1.28	76.7	77.8	75.0	82.4	69.2
Lipid	0.45 (0.24 to 0.66)	1.02	50.0	50.0	50.0	60.0	40.0
Collagen	0.73 (0.50 to 0.89)	1.10	66.7	66.7	66.7	75.0	57.1
metHb	0.78 (0.54 to 0.93)	1.22	73.3	72.2	75.0	81.3	64.3
ln(a)	0.75 (0.50 to 0.88)	1.20	66.7	66.7	66.7	75.0	57.1
b	0.75 (0.53 to 0.90)	1.17	66.7	66.7	66.7	75.0	57.1

HbO, HHb, and water L/N parameters exhibited good discrimination ability between benign and malignant lesions with AUCs≥0.82. At the threshold specified in Table 4, this resulted in accuracy, sensitivity, specificity, PPV, and NPV of 76.7%, 77.8%, 75.0%, 82.4%, and 69.2% for HbO, HHb, and water L/N parameters. MetHb L/N exhibited moderate discrimination ability between the lesion types with an AUC=0.78, corresponding to 73.3% accuracy, 72.2% sensitivity, 75.0% specificity, 81.3% PPV, and 64.3% NPV at the threshold specified in Table 4.

#### 3.2.2 Absolute parameters

**Fig. 6:**
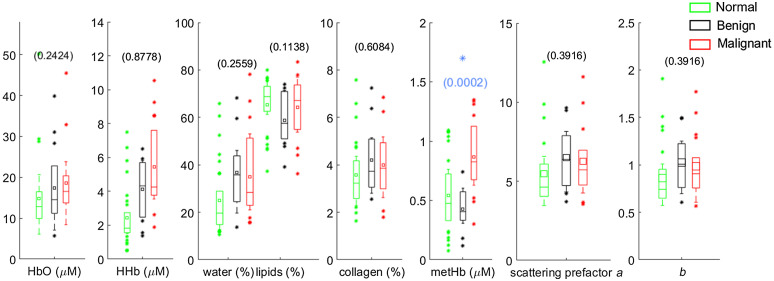
Box plots of absolute measured concentrations in HbO, HHb, water, lipid, collagen, metHb, ln(a), and b in the groups of benign lesions, malignant lesions and normal tissue. Mean value in each group is indicated with a square. Boxes show the median and the 25th and 75th percentiles. Whiskers show the SD, and * represents the outliers. The blue * indicates a statistically significant difference between the benign and malignant groups (Wilcoxon rank Sums test, p-value provided).

Absolute DOS parameters for benign and malignant cases as well as normal tissue are summarized in Fig. 6. The absolute values of scattering parameters and basis chromophores, except HHb, fall in a similar range as the results reported in the original study of only the premenopausal subjects.^21^ The HHb concentrations reported in Ref. 21 were higher for both benign and malignant lesions compared with our results, where some of the HHb content may have been attributed to metHb. The collagen content of malignant lesions (4.2±1.3%) is similar to that observed in a prior study (2.76% observed by Zhao et al.^43^). As expected, collagen and metHb had much lower concentrations compared with the major absorbers (HbO, HHb, water, and lipid). Though the absolute concentration of metHb is small (0.43±0.18  μM for benign versus 0.87±0.32  μM for malignant vsersu 0.54±0.30  μM for normal tissue), metHb was the only statistically significant absolute parameter out of the eight that separated malignant and benign lesion with a p-value of =0.0002, which is <0.00625, the Bonferroni corrected significance level. Moreover, the absolute metHb concentration in normal tissue is comparable to the metHb values observed in benign lesions.

**Fig. 7:**
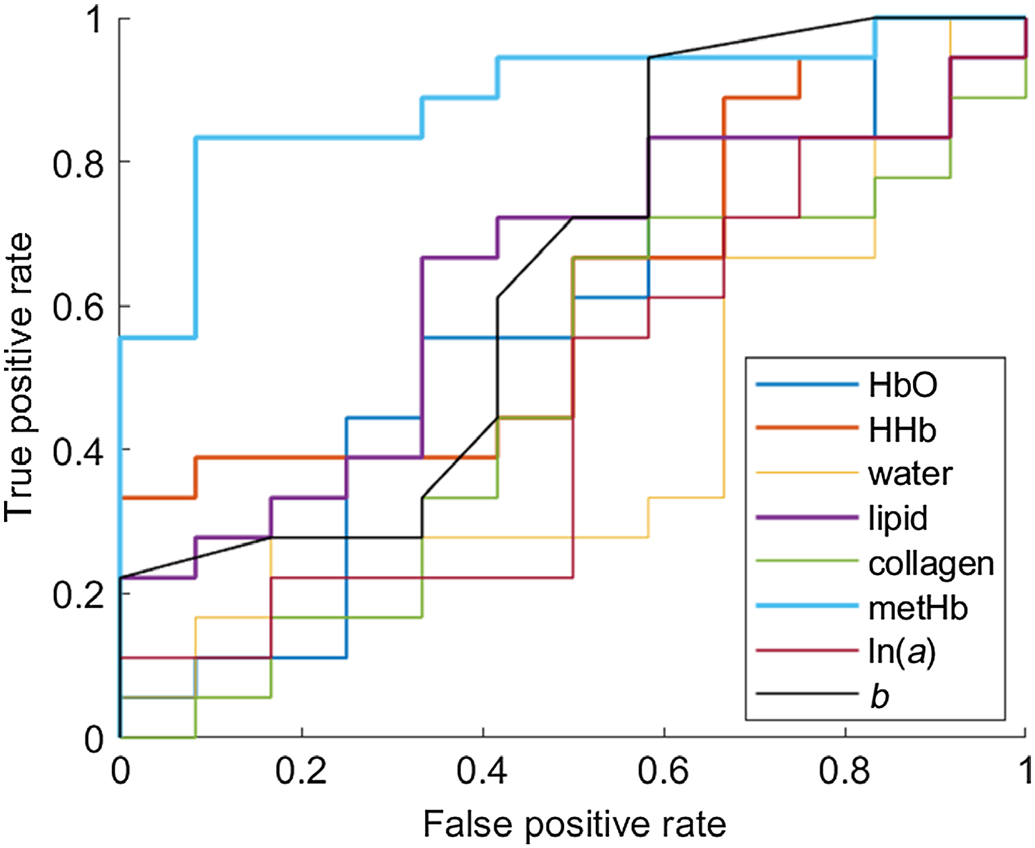
ROC curve of absolute concentrations of DOS parameters.

AUC of all absolute parameters have been shown in Fig. 7 and summarized in Table 5. Accuracy, sensitivity, specificity, NPVs, and PPVs of the absolute parameters at an optimum threshold have also been summarized in Table 5.

**Table 5:** AUC, 95% confidence interval and accuracy, sensitivity, specificity, PPV, and NPV at the specified threshold for absolute parameters.

Absolute parameter	AUC (95 % CI)	Threshold	Accuracy	Sensitivity	Specificity	PPV	NPV
HbO	0.58 (0.34 to 0.80)	15.4 μM	56.7	55.6	58.3	66.7	46.7
HHb	0.63 (0.41 to 0.80)	4.3 μM	50.0	50.0	50.0	60.0	40.0
Water	0.43 (0.21 to 0.66)	31.7%	33.3	33.3	33.3	42.9	25.0
Lipid	0.63 (0.39 to 0.82)	61.7%	66.7	66.7	66.7	75.0	57.1
Collagen	0.47 (0.26 to 0.70)	3.9%	50.0	50.0	50.0	60.0	40.0
metHb	0.89 (0.70 to 0.97)	0.6 μM	83.3	83.3	83.3	88.2	76.9
ln(a)	0.47 (0.26 to 0.71)	5.9	50.0	50.0	50.0	60.0	40.0
b	0.47 (0.24 to 0.68)	0.9	50.0	50.0	50.0	60.0	40.0

Among all absolute parameters, only metHb demonstrated discrimination ability between benign and malignant lesions with an AUC of 0.89 (95% CI 0.70 to 0.97). At the specific threshold of 0.6  μM, this resulted in accuracy, sensitivity, specificity, PPV, and NPV of 83.3%, 83.3%, 83.3%, 88.2%, and 76.9% for metHb concentration. Furthermore, absolute metHb was identified as the best predictor of malignancy exhibiting the highest AUC, accuracy, sensitivity, specificity, PPV, and NPV among all DOS parameters considered in this study (Tables 4 and 5).

The results from the multiple linear regression to examine how each absolute parameter differs by lesion category are summarized in Table 6, adjusting for age and menopausal status. The results indicate that among all absolute DOS parameters, only metHb demonstrated statistically significant difference between the benign and malignant lesion categories (p-value =0.007). Specifically, the metHb level in the malignant group is about exp(0.66)=1.93 folds of that in the benign group, with the same age and menopausal status between the two groups. The results provide further evidence on metHb being a potential robust discriminator for predicting benign versus malignant lesions.

**Table 6:** Regression coefficient estimation associated with lesion category (malignant versus benigh) with std err and p-value in the regression on each absolute parameter.

	HbO	HHb	Water	Lipids	Collagen	metHb	ln(a)	b
Estimate	0.46	0.46	0.32	-0.10	0.08	0.66	0.26	0.24
Std err	(0.22)	(0.24)	(0.18)	(0.08)	(0.17)	(0.23)	(0.13)	(0.12)
p-value	0.043	0.064	0.091	0.230	0.639	0.007	0.059	0.062

## 4 Discussion

Women with radiographically dense breasts are not only at an increased risk for breast cancer but also that a tumor will be occult on mammogram.^44^^,^^45^ Supplemental ultrasound screening have shown to increase the number of cancers detected. However, it doubles the number of unnecessary biopsies and comes with a substantial risk of false positives, leading to many benign biopsy results.^6^^,^^7^ Other imaging approaches used to examine lesions in dense breasts include magnetic resonance imaging, 3D mammography (tomosynthesis), and positron emission tomography.^46^^–^^49^ However, their high cost and limited accessibility preclude them from being routinely applied. Therefore, there is a clinical need for a more accessible adjunct imaging tool that could improve discrimination of benign from malignant lesions and reduce the number of unnecessary biopsies in young women.

Noninvasive DOS is a functional imaging technique that has shown promise in distinguishing benign and malignant breast lesions. DOS studies have reported the presence of small STC, which are not accounted for by major absorbers such as HbO, HHb, water, and bulk lipids,^29^ that are significantly higher in malignant compared with benign lesions.^20^^,^^21^^,^^29^^,^^30^ Although the biochemical origin of STC signatures remains unknown, they have been hypothesized to originate from changes in molecular disposition measured as spectral shifts in water and lipid peaks, and from contributions of minor absorbers (such as hemoglobin by-products and collagen).^21^^,^^29^ Keeping these observations into account, we decided to directly investigate subtle contributions to breast lesion absorption by quantifying collagen and methemoglobin along with the major tissue absorbers (HbO, HHb, water, and lipids).

It was observed that the inclusion of collagen and metHb improves the quality of absorption fit suggesting that there are contributions of other chromophores not accounted for in the basis spectra fit. The improvement in the quality of spectral fit between the linear combination of absorbers and the measured lesion absorption spectrum with the addition of collagen and metHb was evaluated via statistical measures such as RMSE and $R_{a}^{2}$. The increase in $R_{a}^{2}$ value (Table 3) and corresponding decrease in RMSE value (Table 3) validates the improvement of the spectral fit with inclusion of the additional chromophores.

L/N ratios of HbO, HHb, water, (for the first time) metHb, and absolute concentration of metHb were observed to be significantly higher in malignant lesions compared with benign lesions. Specifically, of the optical biomarkers examined, metHb concentration was the most significant parameter for lesion discrimination, with an AUC of 0.89 (95% CI 0.70 to 0.97). Increased HbO, HHb, and water in malignant lesions compared with benign lesions are consistent with tumor volume being characterized by high vascularization, increased perfusion, metabolism, and cellularity. Elevated blood content in malignant versus benign lesions have been previously observed by several other groups using different DOS technologies.^14^^,^^21^^,^^23^^,^^28^ Higher water content in malignant lesions was also reported in other DOS studies.^10^^,^^21^ As far as other constituents of breast tissue are concerned, higher collagen, scattering amplitude, and power L/N values were observed, together with slightly lower lipid L/N values for malignant lesions compared with benign lesions. Elevated normalized collagen content in malignant lesions has been observed by other groups.^23^^,^^43^ Of note, we have corrected our statistical analyses for multiple comparisons, which has not always been done in prior related studies.

Importantly, our work suggests that metHb content is a promising discriminator of malignant and benign lesions, having exhibited the highest confidence (p=0.0002, Fig 6, AUC=0.89, Table 5) among all optical parameters examined in this study. Using a threshold level of 0.6  μM, absolute metHb was able to discriminate benign from malignant lesions with 83.3% accuracy, 83.3% sensitivity, 83.3% specificity, 88.2% PPV, and 76.9% NPV (Table 5). These results are comparable to the performance characteristics of normalized basis absorbers reported in other DOS studies.^21^ However, contrary to those studies, metHb has demonstrated similar diagnostic capabilities in an absolute form.

To the best of our knowledge, breast lesion metHb concentration has not been investigated using an *in-vivo* imaging technique before, though differences were observed between normal and oral cancer tissues with diffuse reflectance spectroscopy.^50^ Increased metHb has also been shown to correlate with higher proliferation in cancer cells.^50^^,^^51^ Methemoglobin is a hemoglobin derivative found in tissue in which the iron moiety of deoxyhemoglobin is in the ferric state rather than in ferrous state.^50^ Hemoglobin oxidized to metHb has been associated with neovascularization and hemorrhage (leaky blood vessels) and may significantly contribute to tumor growth.^50^^,^^51^ Tumour-cell-derived nitric oxide produced during inflammation, hypoxia, and metabolic stress^52^ could also result in the conversion of hemoglobin to metHb.^53^

The major tissue constituents that demonstrated significant differences between benign and malignant lesions in L/N configuration (HbO, HHb, and water) showed no significance when their absolute concentration was considered. This is because L/N ratios account for high interpatient variability of breast composition.^31^^,^^32^ On the other hand, metHb demonstrated higher significance in lesion characterization in its absolute form (p=0.0002, Fig. 6) compared to its L/N configuration (p=0.0052, Fig. 4). MetHb is observed to be less variable on an absolute scale within lesions but more variable when considered as a ratio. The low and variable metHb concentration in normal tissue (0.54±0.30  μM) could also result in significant variability in the L/N ratio. This is because the physiological conditions for hemoglobin to be oxidized to formulate metHb such as abnormal vasculature showing leaky walls and hemorrhage is a common feature of tumors^51^^,^^54^^,^^55^ and are not likely present in the healthy normal tissue. Therefore, metHb is more relevant as an absolute quantification within lesions, which also makes metHb a unique marker of malignancy, as it does not require any measurement of the normal tissue.

The mean age between the benign (47 years) and malignant (53 years) groups are notably different, and the proportion of malignant lesions is significantly higher in postmenopausal subjects (87.5%) compared to premenopausal subjects (52.6%), implying that age and menopausal status are potential confounders in lesion discrimination. The regression results (Table 6) indicated that among all absolute DOS parameters only metHb demonstrated statistically significant difference between benign and malignant lesion categories (p-value=0.007) after correcting for age and menopausal status. This eliminates the concern of confounding factors in the discriminant power of absolute metHb.

Our study has several limitations. First, this was a retrospective analysis of data collected from a relatively homogeneous Korean population. Another limiting factor of this study is that most of the postmenopausal subjects (seven out of eight) had malignant lesions. Future studies should include more cases that achieve a wider distribution of subject characteristics such as age and menopausal status. Furthermore, the average lesion size for malignant lesions in this study was larger than the benign lesions; 2.23 versus 1.63 cm, respectively (Table 2). Partial volume artifacts in DOS may result in an underestimation of L/N and absolute variable values in smaller lesions versus larger lesions.^56^ Furthermore, at comparable lesion depths, a larger lesion is more likely to be probed by the diffuse light field compared with a smaller lesion.^21^ The prior study^21^ explored this potential confounder by analyzing a subset of lesions with similar size and depth. Although, the study found comparable results between the lesion subset and the complete population dataset, they also reported loss of statistical differentiation in a few DOS parameters due to the smaller subgroup dataset. The effect of lesion size, volume, and depths on the estimation of the optical properties and constituent L/N values and absolute concentrations warrants further investigation.

## 5 Conclusions

Quantitative broadband DOS was used to characterize 18 malignant and 12 benign human breast lesions *in vivo*. We examined differences in tissue normalized L/N and absolute concentrations of HbO, HHb, water, lipids, collagen, and metHb as well as scattering parameters (ln(a) and b). Significant differences were observed between malignant and benign groups for HbO L/N (p=0.0014), HHb L/N (p=0.0006), water L/N (p=0.0016), metHb L/N (p=0.0052), and metHb concentration (p=0.0002). Among all parameters investigated, absolute metHb concentration was determined to be the best predictor of malignancy, with an AUC of 0.89 (95% CI 0.70 to 0.97). The regression analysis also indicated that absolute metHb maintained the statistically significant difference between lesion categories after correcting for potential confounding factors including age and menopausal status (p=0.007). MetHb concentration showed the most significant difference between the lesion types, suggesting it to be an important optical biomarker of breast cancer. Until now, metHb has not been examined in breast lesions, but its contribution to noninvasive lesion characterization might prove vital. Future studies should include investigation of metHb variation among additional lesion types that involves more diverse lesion classifications, such as cysts, fibroadenomas, and other benign lesion types.
